# An old foe on peculiar paths: severe falciparum malaria in a Syrian refugee, possibly infected during migrant smuggling from Türkiye to Germany

**DOI:** 10.1007/s15010-023-02042-7

**Published:** 2023-05-24

**Authors:** Jonathan F. Brozat, Miriam Haverkamp, Philipp Hohlstein, Jule K. Adams, Theresa H. Wirtz, Hanna R. Klingel, Susanne Hürtgen, Karim Hamesch, Tony Bruns, Christian Trautwein, Samira Abu Jhaisha, Alexander Koch

**Affiliations:** 1 Department for Gastroenterology, Metabolic Disorders and Intensive Care Medicine, University Hospital RWTH Aachen, RWTH Aachen, Pauwelsstraße 30, 52074 Aachen, Germany; 2 Department of Infection Control and Infectious Diseases, University Hospital RWTH Aachen, RWTH Aachen, Aachen, Germany; 3 Laboratory Diagnostic Center, University Hospital RWTH Aachen, RWTH Aachen, Aachen, Germany; 4 Department of Gastroenterology, Rhein-Maas Hospital, Würselen, Germany

**Keywords:** Falciparum malaria, Migrant smuggling, Refugee,, Transmission, Post-artesunate delayed hemolysis

## Abstract

Infectious diseases and their imperative awareness gain major relevance through global warming and multi-continent refugee crises. Here, we demonstrate the challenges of malaria diagnosis, disease course, and treatment, including post-artesunate hemolysis in a Syrian refugee with severe *falciparum* malaria, most probably infected during migrant smuggling from Türkiye to Germany.

## Introduction

*Plasmodium falciparum*, the pathogen causing malaria tropica, remains one of the most potent and deadliest of parasites known to humankind, causing an estimated 619,000 in 2021 [1]. *P. falciparum* predominantly occurs in the (sub)tropical zones of sub-Saharan Africa, Asia, and Latin America.

To complete their life cycle, any of the known *plasmodia* infecting humans, need to develop within blood-feeding insects from gametes to sporozoites over ten to 18 days before becoming infectious and being injected into the human blood stream. *Plasmodia* subsequently infect human liver cells (schizonts), followed by erythrocytes (trophozoites), causing both hepatocellular damage and sequestration of mature-staged red blood cells. Severe malaria is thus facilitated by microcirculatory congestion and organ failure [2]. The incubation time in immunologically naïve individuals is between six and 30 days. Clinical presentation can vary depending on the *plasmodia*, from the initial presentation with (mild) periodic fever to signs of cerebral malaria and full-scale multi-organ failure.

In humans, *plasmodia* are only transmitted through the bite of an infected endemic female *Anopheles* mosquito [3]. Patients with *P. falciparum* malaria are thus exceedingly rare in both continental Europe and North America. Cases with symptomatic malaria are mainly imported through travelers and immigration. Autochthonous *P. falciparum* transmission has sporadically been witnessed through ‘backpack mosquitoes’, in the vicinity of airports, or even in hospitals [4, 5]. Identically, *P. vivax* was considered eradicated in Europe, but has since been re-introduced to Greece via imported cases [6].

As transmissions seldomly occur outside of the tropical zones, daily experience with diagnostics, disease course, and treatment options in *plasmodium* infections is dangerously limited within colder regions and hospital personnel without access to infectious disease specialists.

Fueled by rising temperatures and large-scale refugee crises, incidence rates of infectious diseases will increase. It is thus highly necessary to report on and reshape awareness for (tropical) infectious disease, preventing misdiagnosis -and treatment.

## Case report

Here, we report on the case of a 38-year-old female, who was transferred from an outside hospital to our intensive care unit (ICU) with severe malaria. Four days prior she had developed fatigue, jaundice, headaches, and exertional dyspnea. Before admission, the patient reported subjective fever, but the temperature was not elevated in the emergency department. Chest X-rays did not demonstrate pathologies and PCRs for respiratory viruses, such as influenza or SARS-CoV-2 remained negative.

Upon transfer to our service, laboratory parameters demonstrated hypoglycemia, severe hemolysis and thrombopenia as well as acute hepatopathy with a total serum bilirubin of 8.2 mg/dL (equals 140 μmol/L, reference < 1.2 mg/dL), conjugated bilirubin of 6.7 mg/dL (equals 115 μmol/L, reference < 0.3 mg/dL), and an international normalized ratio of 1.32. Serum alanine aminotransferase (ALT) was 102 U/L (reference < 35 U/L) and aspartate aminotransferase (AST) was 76 U/L (reference < 35 U/L). Additionally, the patient developed elevated inflammatory markers with a C-reactive protein (CRP) of 154 mg/L (reference < 5 mg/dL) and procalcitonin (PCT) of 4.7 ng/mL (reference < 0.5 ng/mL). No leukocytosis was present, and hemoglobin at presentation was 16 g/dl (reference 11.2–15.7 g/dL) and decreased to 13 g/dl within 16 h, with lactate dehydrogenase (LDH) initially at 471 U/L (reference < 250 U/L). Renal function remained within the normal range.

**Fig. 1:**
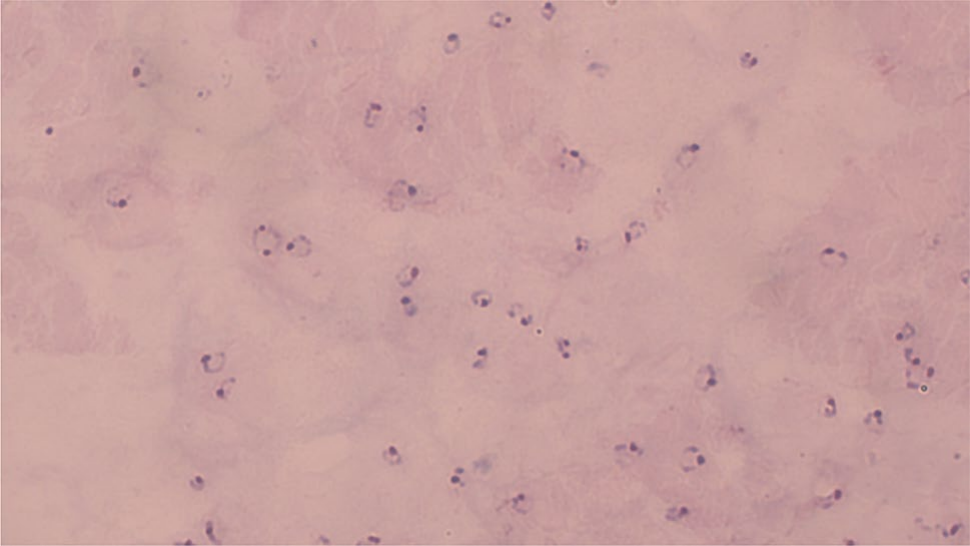
Blood smear, Giemsa stain. Histological features of *Plasmodium falciparum* malaria include ring-shaped early trophozoites (“ring stage’) and multiple infected red blood cells. Schizonts, Maurer’s clefts, and typically elongated, crescent-shaped gametocytes are missing in this blood smear, as late trophozoites and schizonts are usually not seen in the periphery in falciparum malaria and gametocytes usually only occur after seven days

During a microscopic examination of a routine blood smear, performed to rule out hematologic malignancies as the cause, the red blood cells (RBCs) showed a significant number of ring-shaped early trophozoites (Fig. 1). The thick smear confirmed malaria, with the rapid diagnostic test (RDT) for *P. falciparum* being highly positive [7]. Parasitemia at presentation was 2% (20 per 1000 RBCs).

The patient was transferred to our university hospital with early signs of cerebral malaria, such as headaches, somnolence, and a high fever and thus directly admitted to our intensive care unit. Within hours she developed severe hypotension requiring vasopressor therapy. The initial regimen of artemether/lumefantrine was escalated to intravenous artesunate (IVAS) when we received further results of the thin smear with a parasitemia of 13% (130 per 1000 RBC) (Fig. 1) [2, 8]. Following intravenous artesunate treatment, parasitemia decreased to < 1% within 24 h and the patient stabilized. Intravenous artesunate was thus discontinued and artemisinin-based combination therapy (ACT) with artemether–lumefantrine was re-initiated. The patient was transferred to standard care, where laboratory parameters initially continued normalizing.

Ten days after receiving artesunate treatment, the patient experienced significant hemolysis, which became increasingly severe. The level of LDH peaked at 2013 U/L on the 15th day post-treatment. Hemoglobin decreased to 5.9 g/dL and the patient needed transfusions of erythrocyte concentrates. Blood smears remained negative for *plasmodia* excluding recrudescence or drug resistance. We diagnosed severe post-artesunate delayed hemolysis (PADH), an uncommon but in many cases self-limiting adverse event in IVAS treatment that mainly occurs within 14 days of intravenous administration [9].

In this case, hemolysis was self-limiting; the patient showed significant improvement and was discharged 25 days after the onset of symptoms.

The Syrian patient arrived in Germany only ten days prior to the presentation. She had fled from northern Syria on foot to a rural village in Türkiye where she remained for several days. From there she went to Istanbul where she was picked up by traffickers. The patient and other migrants, possibly from malaria-endemic countries, were loaded into the back of a van without windows and driven through Europe. The trip took four days and involved one transfer into another van. Upon arrival in Germany, all migrants were instructed to delete their phone records and were provided with train tickets to a destination of their choice within Germany.

## Discussion

*P. falciparum* is not endemic to Northern Syria, Türkiye, or the Balkans. Due to the short time span of incubation for *P. falciparum*, a transmission prior to departure from Syria seems highly unlikely [10]. Until the recording of cases stopped due to the civil war, Syria was only known for the habitual distribution of *P. vivax* [11]. The patient denied an increase in malaria cases or children under five deaths prior to her departure which could have indicated an undetected Malaria outbreak in the region where the patient originated. The characteristics of the case lead to our hypothesis that transmission of *P. falciparum* in this patient might have occurred during the transportation to Europe, possibly through an infected mosquito hidden in the bags of another migrant from a *P. falciparum*-endemic country, travelling in the same van. As all migrants were instructed to delete their phone records and were heading to different locations within Germany, tracking and treating other infected individuals proved impossible, thus highlighting that especially refugees are vulnerable to a delayed diagnosis. A global movement of people and goods in combination with global warming poses a risk for (re)introduction of new or locally extinct diseases, as demonstrated by the autochthonous malaria transmission in Greece, starting in 2009, as well as Italy in 2017 [4, 6, 12]. As accurate and timely tracking will remain difficult, it is paramount to rethink diagnostic strategies, entomological surveillance, and early detection of cases of tropical diseases thought to be (locally) eliminated [13].

Apart from highlighting the unexpected circumstances of transmission within the challenges of the modern world, this case stresses several notable aspects of clinical management of (a)typical falciparum malaria: The necessity of quick differential diagnoses to different and unexpected forms of hemolysis and hepatopathy, the management of high parasitemia with ACT and IVAS, as well as the severity of post-artesunate delayed hemolysis.

Risk factors for PADH include non-immune travel status, high initial parasitemia, and cumulative IVAS dose of > 9.5 mg/kg, of which only the parasitemia applied to our patient [9]. PADH occurs rarely and only after RBCs have been cleared of parasitic infection (‘pitting’) and malaria symptoms alleviated [14]. Somewhat irritatingly for physicians, the direct antiglobulin (Coombs) test (DAT) might be positive in up to 50% of PADH-cases, possibly a symbol for immune activation, rather than direct anti-erythrocytes antibodies [15]. PADH pathology is believed to rely on shortened RBC life spans post-infection, conveying a delayed clearance [9, 14].

Patients treated with IVAS require routine laboratory checks for four to six weeks following treatment. Optimally, monitoring should include hemoglobin, reticulocyte count and production index (RPI), lactate dehydrogenase, haptoglobin, and total bilirubin.

Globally, the debate on IVAS treatment and its clinical consequences is of high relevance. After isolation in China in the mid-1970s, intravenous artesunate has only been approved for use by the U.S. Food and Drug Administration (FDA) in May 2020, ending a period without FDA-approved intravenous malaria medication since the discontinuation of quinine in 2019. In Europe, the European Medicines Agency (EMA) approved artesunate in November 2021 for marketing with additional monitoring [16]. Knowledge of adverse effects amid large-scale refugee movements becomes thereby increasingly relevant.

This case impressively demonstrates the necessity for increased awareness towards tropical diseases as a challenge for physicians in an ever more globalized world. Despite the lack of a clear exposure, this patient suffered from *P. falciparum* malaria, which could have been easily missed, and could have been fatal, had the parasitemia not been this high by the time a manual blood smear was done, or no manual blood smear had been ordered.

## Data Availability

Data are available from the corresponding author on reasonable request.
